# Did She or Didn't She? Perceptions of Operative Status of Female Genitalia

**DOI:** 10.1093/asj/sjae130

**Published:** 2024-06-13

**Authors:** Daniel C Sasson, Gemma Sharp, Otto J Placik

## Abstract

**Background:**

Although extensive research has explored why women undergo labiaplasty, little attention has been paid to societal and professional abilities to distinguish between altered and unaltered labia, impacting both patient concerns and broader societal perceptions.

**Objectives:**

This study aimed to evaluate the accuracy of the general public and healthcare professionals in identifying labiaplasty and to pinpoint the misconceptions driving their perceptions. The goal was to inform more effective patient counseling strategies and challenge existing stigmas around cosmetic genital surgery.

**Methods:**

The authors conducted an online survey of 511 lay adults and a group of 21 gynecologists and aesthetic vulvar surgeons. The survey assessed the participants’ ability to detect labiaplasty from images, focusing on aesthetic appearance, hair patterns, and size. The analysis involved Pearson correlation and *Z*-tests to compare perceptions against actual operative status.

**Results:**

Analysis of the survey findings revealed a pronounced difficulty among participants in accurately discerning labiaplasty, with neither group showing a significant ability to identify surgical alterations. Misinterpretations were notably influenced by expectations of aesthetic appearance, with 49% associating an “odd” or “fake” look with surgery, and hair and size misconceptions also misleading respondents. Additionally, 20% of participants mistakenly related surgical changes to gender-affirming surgery or female genital mutilation.

**Conclusions:**

The study highlights a gap in the ability of both the general public and medical professionals to accurately identify labiaplasty, pointing to a broad misunderstanding of cosmetic genital surgery's visual outcomes. Addressing these misconceptions through targeted education could substantially improve patient counseling and help dismantle the stigmas associated with labiaplasty.

**Level of Evidence: 3:**

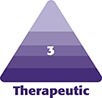

Extensive research has delved into the motivations behind women seeking labiaplasty, yet comparatively little attention has been given to exploring their apprehensions or anxieties regarding the procedure.^1-8^ Although prioritizing functional objectives is crucial, surgeons frequently highlight the desire to achieve a conventional aesthetic appearance as a driving factor for patients considering labiaplasty.^7,9-11^ Obtaining these outcomes necessitates a precise understanding of anatomy and surgical techniques.^12^ Surgeons have been advised to inquire about their patients’ postoperative expectations regarding projection, length, color, size, and other relevant factors.^13^ This recommendation aims to better fulfill patients’ desires and achieve satisfactory outcomes. Interestingly, women who have requested labiaplasty report experiencing similar levels of comments about their previous appearance as women who have not sought the procedure.^14^ Although achieving a natural result may appear straightforward, patients have voiced concerns and anxiety about potential detection of surgical alterations to the labia by future partners during intimate interactions.^3,15-18^ Recent findings suggest that women undergoing labiaplasty worry about how their partners may perceive any scarring, fearing implications such as being mistaken for transgender individuals who have undergone gender-affirming genital surgery.^19^ This is supported by surveys indicating that this procedure carries a societal stigma disproportionate to other cosmetic surgical procedures.^6,20^ These concerns are possibly unwarranted in light of numerous studies affirming the safety and cosmetic improvements of labiaplasty.^21-25^

The aim of this study was to survey the general population to ascertain whether individuals can discern between operated and unoperated labia. Our hypothesis posits that the lay public will be unable to differentiate between the two.

In addition, we compared these findings with the perceptions of gynecologists and surgeons who identify as aesthetic vulvar surgeons, and further hypothesize that physicians who specialize in aesthetic vulvar procedures will demonstrate greater accuracy in discerning between operated and unoperated labia compared to the lay public, given their expertise and daily exposure to this anatomy.

## METHODS

All procedures followed were in accordance with the ethical standards of the responsible committee on human experimentation (institutional and national) and with the Helsinki Declaration of 1975, as revised in 2008. Informed consent was obtained from all patients for being included in the study.

An online survey eliciting opinions of vulvar appearance and surgical status was utilized (Appendix). Respondents were consecutive English-speaking individuals 18 years of age or older from all educational backgrounds who could complete a visually based CAPTCHA challenge.^26^ The survey was crowdsourced online via Amazon Mechanical Turk (MTurk, Seattle, WA) for 3 weeks in December 2023. Respondents were compensated $1.00 for completing this survey. Additional responses were gathered through personal networks from US-based gynecologists and surgeons who identify as aesthetic vaginal surgeons. Responses were deidentified automatically to maintain maximum confidentiality. Although “vulva” (and specifically, the labia minora) is a technically accurate term for the external genitalia that are operated upon, we chose to use “vagina” throughout the survey as it is more commonly understood by lay people.^27,28^

### Study Design and Distribution

After collecting demographic information, respondents were asked to disclose their personal history of cosmetic surgery, as well as their opinions regarding the acceptability of vulvar cosmetic surgery for consenting adults. Subsequently, a randomized series of images depicting 10 women—5 who underwent surgical intervention (labiaplasty) and 5 who did not, in both lithotomy and standing positions—was presented. Randomization was performed with a random number generator to ensure that the selection process for the survey images was unbiased.^29^ Express consent was obtained for all images utilized. For each woman, respondents were tasked with determining whether surgical intervention had occurred, assessing the natural appearance of the vulva, and evaluating its aesthetic appeal. Finally, respondents were prompted to choose from a list of 4 to 7 reasons or provide their own rationale for their determinations regarding both surgically altered and natural-looking vulvas.

### Statistical Analysis

Descriptive statistics were computed for demographic data. The Pearson correlation coefficient (*r*) between “natural” and “aesthetic” ratings was calculated. Additionally, a *Z*-test of mean “natural” and “aesthetic” scores by operative status was conducted. Furthermore, a 2-sample heteroscedastic *t*-test for differences in respondent perception of operative status by actual operative status was performed. Significance was set at *P* < .05. All statistical analyses were carried out with the SciPy Python library (version 1.11.1).^30^

## RESULTS

### Layperson Demographics

In total, 511 lay adults completed the survey. Respondents’ mean [standard deviation] age was 38.7 [11.2] years. Of this total, 245 (48%) were female, 264 (51.6%) male, and 2 (0.4%) nonbinary/genderqueer/agender; 230 (45%) responded affirmatively, stating that they had undergone cosmetic surgery themselves; 384 (75%) felt vulvar cosmetic surgery by a consenting adult to be “slightly acceptable” or “acceptable.”

### Layperson Responses

A moderately high correlation was observed between “natural” and “aesthetic” ratings (*r* = 0.66, *P* = .001). Notably, there was no significant difference in “natural” or “aesthetic” ratings based on the operative status of the imaged woman (*P* = .89, *P* = .96, respectively). On average, 62.3% of respondents believed any woman imaged had been operated upon; this proportion was consistent regardless of the actual operative status of the woman displayed (operated, 61.5%; unoperated, 63.1%). The mean proportion who were correct for each woman depicted was 49.2%. There was no significant correlation between respondents’ perception of operative status and the actual operative status of the women (*P* = .81).

In assessing the characteristics attributed to labia perceived as not surgically altered, participants indicated their judgments were influenced by several factors. Specifically, aesthetic appearance was cited by 307 (60%) respondents as a determinant, followed by the quality or type of hair, mentioned by 257 (50%) participants. The appearance of being unoperated was noted by 223 (44%) respondents, and the perception of size, with larger-looking labia being identified by 176 (34%) participants as appearing not surgically altered. Furthermore, 9 respondents (2%) independently indicated that larger-looking labia appear not surgically altered.

For labia perceived as having undergone surgical intervention, participants attributed their assessments to a variety of indicators. The most commonly cited reason was an odd or fake appearance, noted by 252 (49%) participants, followed by a perception of being too perfect, mentioned by 222 (43%) respondents. Aesthetic appearance influenced the judgments of 202 (40%) participants, while the presence of an obvious scar was a factor for 158 (31%) individuals. Hair quality or type was considered by 146 (29%) respondents, and the appearance suggestive of a transgender individual's genitalia was noted by 115 (23%) participants. The perception of resembling female genital mutilation—defined for the purposes of this study as procedures injuring the vagina for nonmedical or noncosmetic reasons—was cited by 100 (20%) respondents. Furthermore, 8 participants (2%) independently indicated that the presence of smaller labia contributed to their assessment.

### Physician Demographics

In total, 10 self-identified aesthetic vulvar surgeons and 11 general gynecologists completed the survey. Respondents’ mean age was 57.4 [9.6] years, and 9 (43%) were female. Of these 21 physicians, 4 (19%) responded affirmatively, stating that they had undergone cosmetic surgery themselves. Among them, 20 (95%) found vulvar cosmetic surgery by a consenting adult to be “slightly acceptable” or “acceptable.”

### Physician Responses

A low correlation was observed between “natural” and “aesthetic” ratings (*r* = 0.21, *P* = .35). Similarly, there was no significant difference in “natural” or “aesthetic” ratings based on the operative status of the imaged women (*P* = .61, *P* = .21, respectively). On average, 51.9% believed any woman imaged had been operated upon; this proportion varied slightly with regards to actual operative status of the woman displayed (operated, 60.0%; unoperated, 42.8%). The mean proportion who were correct for each woman depicted was 58.1%. Collectively, there was no significant correlation between respondent's perception of operative status and the actual operative status of women (*P* = .28). This remained consistent when respondents were categorized by practice type (aesthetic vulvar surgeons, *P* = .42; general gynecologists, *P* = .19).

In assessing the characteristics attributed to labia perceived as not surgically altered, physicians indicated their judgments were influenced by several factors. Specifically, aesthetic appearance was cited by 10 (48%) respondents as a determinant, and the quality or type of hair, mentioned by 1 (5%) participant. The appearance of being unoperated was noted by 11 (52%) respondents, and the perception of size, with larger-looking labia being identified by zero participants as appearing not surgically altered.

For labia perceived as having undergone surgical intervention, participants attributed their assessments to a variety of indicators. An odd or fake appearance was noted by 4 (21%) participants, and a perception of being too perfect was mentioned by 2 (10%) respondents. Aesthetic appearance influenced the judgments of 11 (52%) participants, while the presence of an obvious scar was a factor for 11 (52%) individuals. Hair quality or type was considered by 1 (5%) respondent, and the appearance suggestive of a transgender individual's genitalia was noted by 1 (5%) participant. The perception of resembling female genital mutilation was cited by 1 (5%) respondent. Furthermore, 2 participants (10%) independently indicated that the presence of smaller labia contributed to their assessment.

### Layperson-Physician Comparison

Although neither lay adults nor physicians accurately determined the operative status of the woman depicted, physicians exhibited a superior ability to do so (58.1% vs 49.2%, *P* < .001). No disparities in detective ability were observed between aesthetic vulvar surgeons and general gynecologists (59.0% vs 57.3%, *P* > .99).

## DISCUSSION

Our hypothesis that the lay public could not discern surgically altered from unoperated labia was supported by this novel survey. Furthermore, the statistical data indicated that physicians, including gynecologists and aesthetic vulvar surgeons, although with slightly superior performance, were similarly unable to distinguish the difference. This aligns with the authors’ clinical observation that women who are hesitant to disclose the procedure to their gynecologist for fear of judgment often report that their surgery remains undetected during subsequent gynecological examinations. Although previous studies have provided documentation to support and allay the fears of women undergoing labiaplasty regarding cosmetic benefits, preservation of sensation, and potential effects on vaginal delivery, these results further support that surgical changes will not be recognized by the general population or by experienced practitioners.^24,31-35^ Surprisingly, surveyed individuals have expressed reluctance to undergo the labiaplasty procedure because of worries that they may be labeled as victims of female genital mutilation or to have undergone gender-affirming procedures.^6,19^ These findings provide valuable insights to counsel patients considering the procedure and address concerns regarding potential detection of surgical alterations by future intimate partners.

Notably, half of lay adults associated a denser hair pattern with natural vulva appearance, underscoring the necessity of incorporating discussions about hair patterns into presurgical counseling to align patient expectations with outcomes that are perceived as natural or unaltered. Hair pattern can be influenced by grooming and aging, factors that are largely independent of labiaplasty. Furthermore, participants erroneously identified labia as unoperated based solely on appearance, which effectively challenges the accuracy of both lay people and physicians in distinguishing between operated and nonoperated labia. This observation reinforces our belief that individuals lack the ability to accurately discern between surgically altered and natural labial appearances.

Moreover, the belief held by 37% of respondents that a larger labial size is indicative of a natural appearance, coupled with their general inaccuracy in identifying surgical modifications, may reflect a selection bias in our study towards samples of operated labia that were conservatively altered, avoiding the production of overly reduced labial sizes postoperatively.

Our findings also reveal concerning societal attitudes towards vulvar appearance postlabiaplasty. The misperception by approximately half of lay adults that an odd or unnaturally perfect appearance indicates surgical modification highlights a stigmatized view of cosmetic genital surgeries. The fact that similar proportions of respondents equated too-perfect appearances with aesthetic enhancements further complicates public understanding and acceptance of these procedures. Notably, around a quarter of respondents mistakenly used hair and scar patterns as indicators of surgical intervention, which proves unreliable.

Alarmingly, 20% of participants associated surgically altered labia with gender-affirming surgery or female genital mutilation, revealing a profound misunderstanding and conflation of cosmetic surgical procedures with gender and cultural practices. These misconceptions warrant a concerted effort towards enhancing public education and awareness regarding vulvar aesthetics, the diverse reasons individuals may choose labiaplasty, as well as gender-affirming surgery. Such initiatives are crucial not only for dispelling myths and reducing stigma but also for fostering a more inclusive and informed dialogue about bodily autonomy and cosmetic surgery.

There are several limitations to this study. Firstly, it does not assess the degree of improvement achieved. However, this aspect has been addressed in a large-scale survey, which demonstrated favorable views of labiaplasty results.^36^ Future studies may consider direct assessment of before-and-after results of those 5 patients operated upon in this study.

Additionally, there exists a selection bias in the inclusion of a limited number of operated and natural (unoperated) individuals, all of whom were US-based patients of varying races, ages, and body types, with surgeries performed by a single surgeon in one practice. This may not be representative of all surgical outcomes, as results may vary among different surgeons. Notably, 4 of the 5 surgical results were achieved using a trim technique, while 1 utilized a wedge technique, which may yield different outcomes. Furthermore, there is a potential bias in selecting unoperated controls, as individuals willing to be photographed for controls may exhibit characteristics such as being happier with their appearance, more groomed, or having fewer insecurities or personal concerns about their genitalia, which may not be representative of the average person disinterested in surgery. Additionally, the term “aesthetic” was defined, which can be subjective and problematic as it encompasses concepts such as beauty, attractiveness, and appeal. Finally, our sample of lay adults may not have been entirely representative of a community sample given their high rates of having undergone cosmetic surgery themselves (45%) and support for cosmetic vulvar surgery (75%). Future research with more diverse samples would be beneficial to confirm these novel and interesting results.

## CONCLUSIONS

Regardless of whether the patient's concerns are aesthetic or functional, it is imperative for practitioners to prioritize alleviating patient distress regarding the procedure.^37^ With this principle in mind, the information gleaned from this study presents value. Specifically, it reveals that neither prospective partners nor medical practitioners experienced in treating female anatomy are able to discern between surgically altered and unoperated labia.

## Supplemental Material

This article contains supplemental material located online at www.aestheticsurgeryjournal.com.

## Supplementary Material

sjae130_Supplementary_Data
