# Supplementary material for: Did She or Didn't She? Perceptions of Operative Status of Female Genitalia
Source: Aesthet Surg J. 2024 Jun 13;44(11):1203–8. doi: 10.1093/asj/sjae130 (PMC11474600; doi:10.1093/asj/sjae130)
Supplement: sjae130_Supplementary_Data [file sjae130_supplementary_data.pdf]

Vaginal Cosmetic Surgery Survey

We are doing a survey to find out if people can tell whether a vagina has undergone cosmetic surgery.

* Indicates required question

Only answers that show thoughtful consideration will receive immediate payment through MTurk. Thank you for your cooperation.

1. MTurk Worker ID *

CAPTCHA

2. How many squares contain bicycles? *

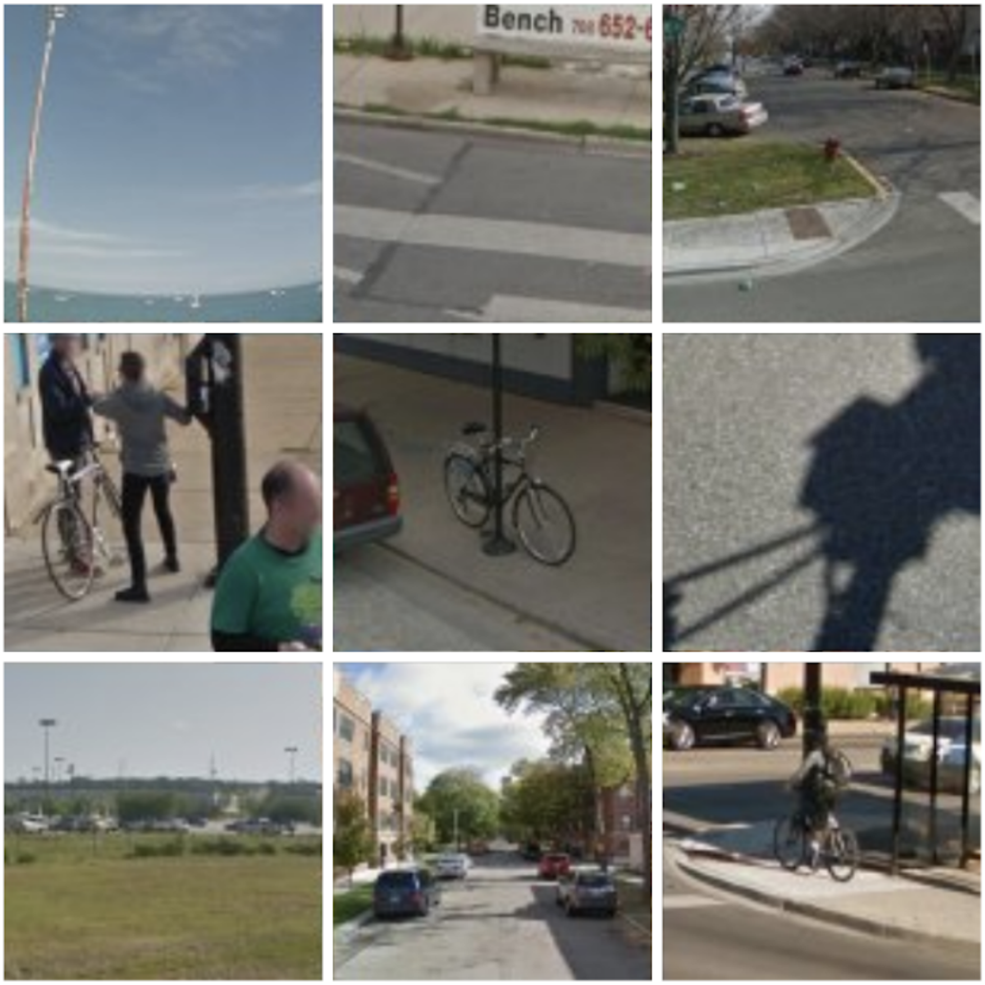

Demographics

3. Age *

4. Gender Identity *

Mark only one oval.

- Male
- Female
- Transgender Male
- Transgender Female
- Non-binary / Genderqueer / Agender
- Another identity not listed above

5. Have you ever had cosmetic surgery on ANY part of your body/face? *

Mark only one oval.

- Yes
- No
- Prefer not to say

6. How do you feel about vaginal cosmetic surgery by a consenting adult? *

Mark only one oval.

- Acceptable
- Slightly Acceptable
- Neutral
- Slightly Unacceptable
- Unacceptable

Picture 1.

SUPINE (LAYING DOWN) STANDING

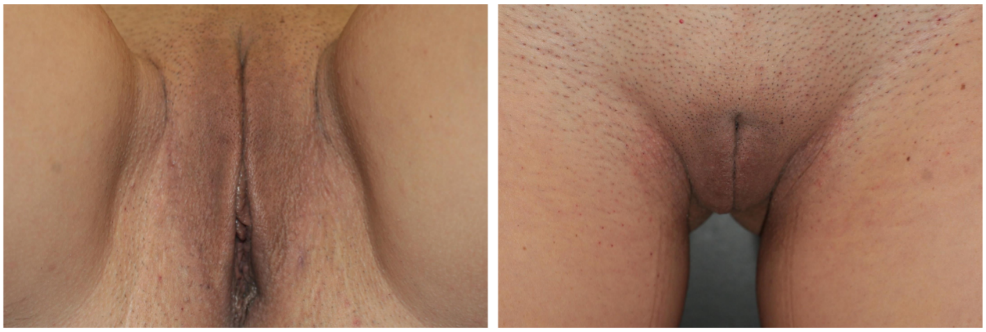

7. Has this person had vaginal cosmetic surgery? *

SUPINE (LAYING DOWN) STANDING

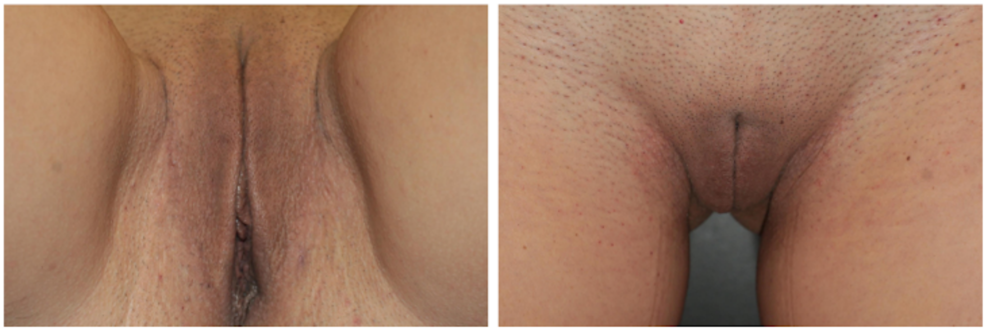

Mark only one oval.

- ⬭ Yes
- ⬭ No

8. Does this vagina look natural? *

SUPINE (LAYING DOWN) STANDING

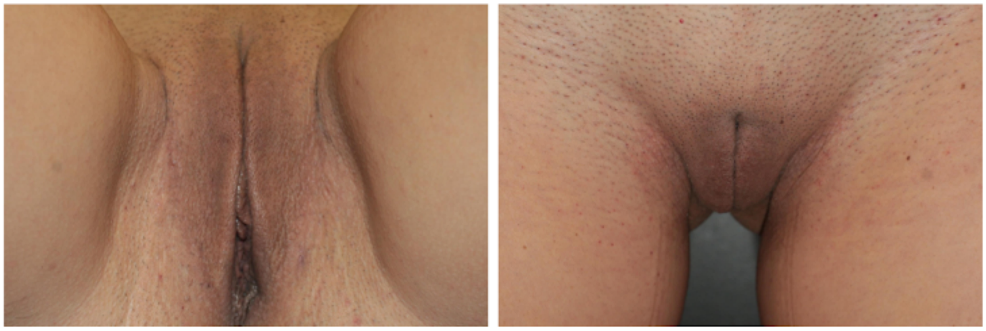

Mark only one oval.

	1	2	3	4	5	
Not	◯	◯	◯	◯	◯	Natural

9. Does this vagina look aesthetic (i.e. beautiful, cosmetic, attractive, or appealing)? *

SUPINE (LAYING DOWN) STANDING

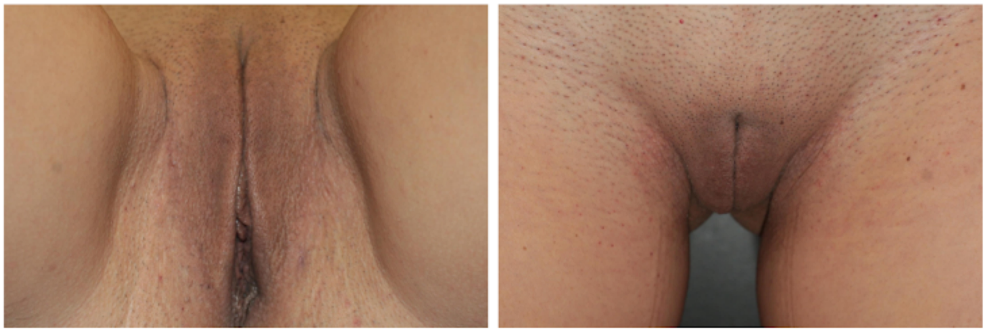

Mark only one oval.

	1	2	3	4	5	
Not	◯	◯	◯	◯	◯	Aesthetic

Picture 2.

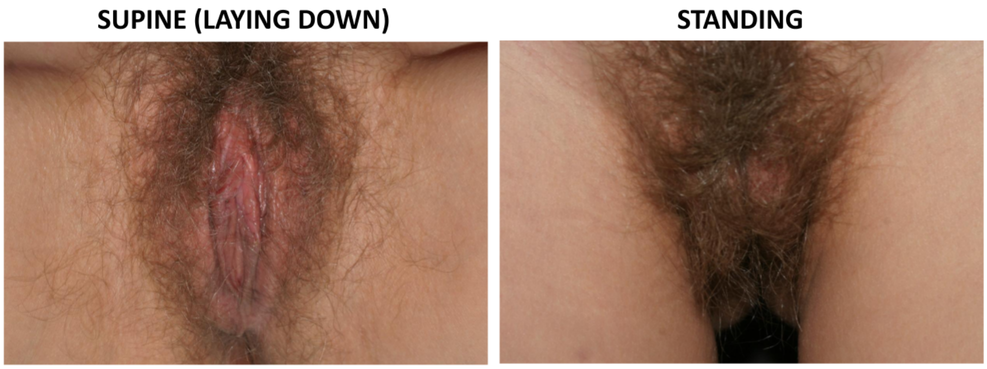

10. Has this person had vaginal cosmetic surgery? *

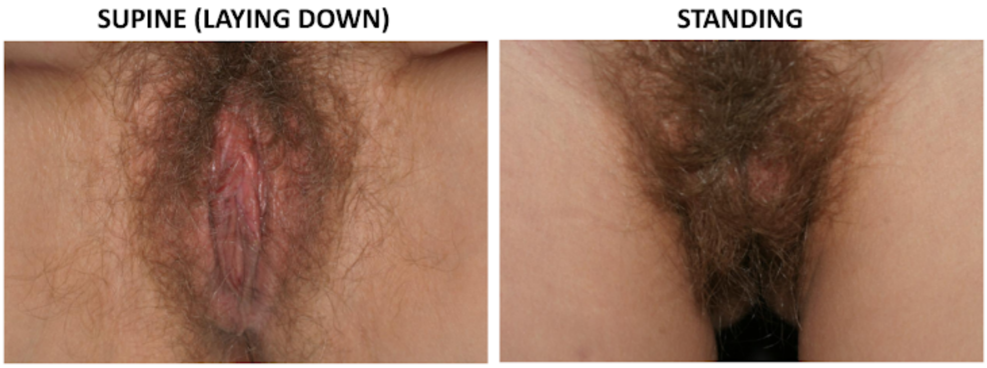

Mark only one oval.

- ( ) Yes
- ( ) No

11. Does this vagina look natural? *

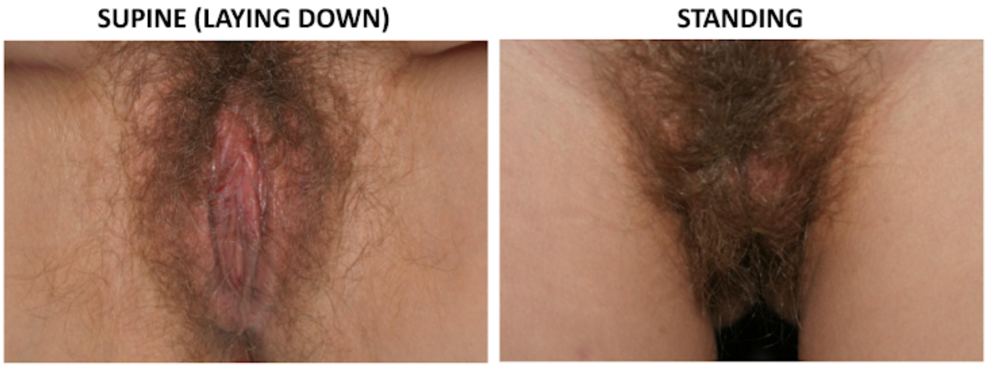

Mark only one oval.

	1	2	3	4	5	
Not	◯	◯	◯	◯	◯	Natural

12. Does this vagina look aesthetic (i.e. beautiful, cosmetic, attractive, or appealing)? *

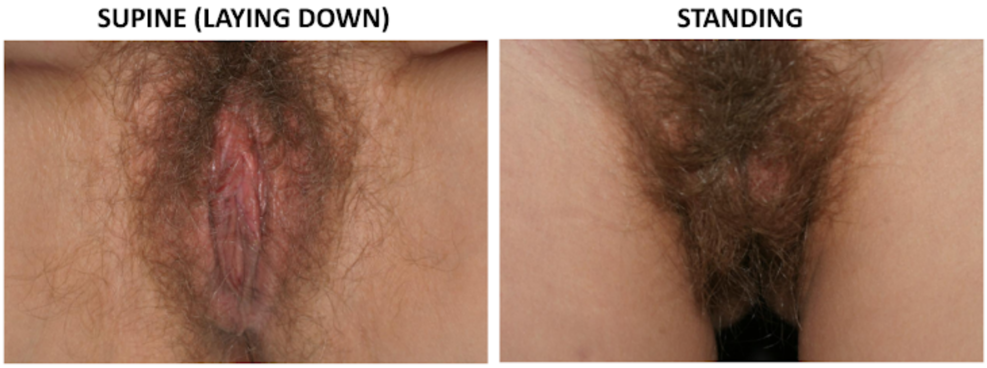

Mark only one oval.

	1	2	3	4	5	
Not	◯	◯	◯	◯	◯	Aesthetic

Picture 3.

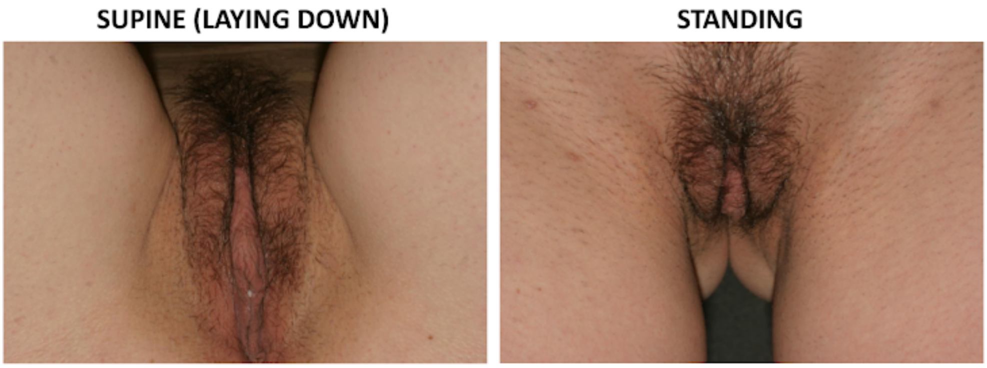

13. Has this person had vaginal cosmetic surgery? *

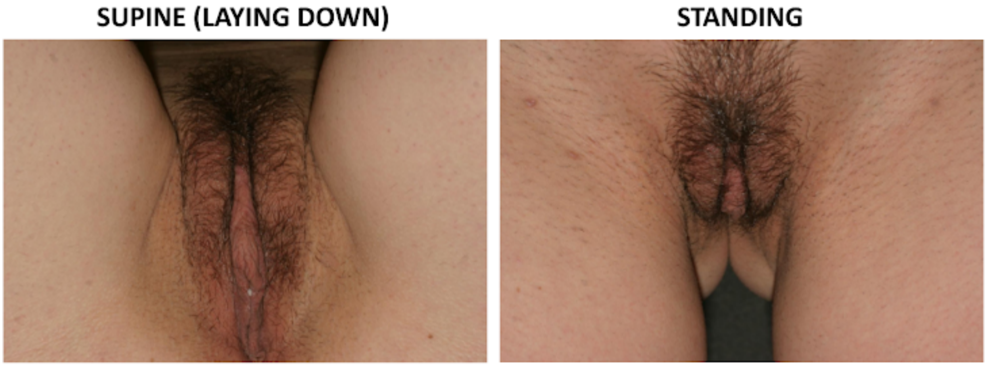

Mark only one oval.

- ⬭ Yes
- ⬭ No

14. Does this vagina look natural? *

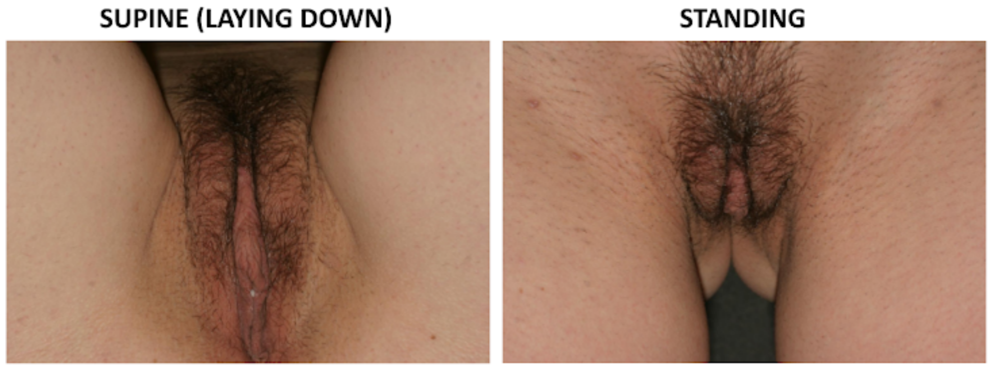

Mark only one oval.

	1	2	3	4	5	
Not	◯	◯	◯	◯	◯	Natural

15. Does this vagina look aesthetic (i.e. beautiful, cosmetic, attractive, or appealing)? *

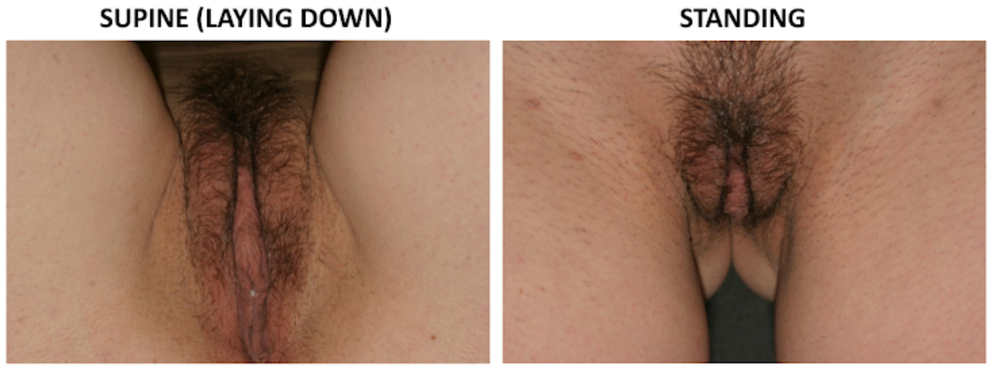

Mark only one oval.

	1	2	3	4	5	
Not	◯	◯	◯	◯	◯	Aesthetic

Picture 4.

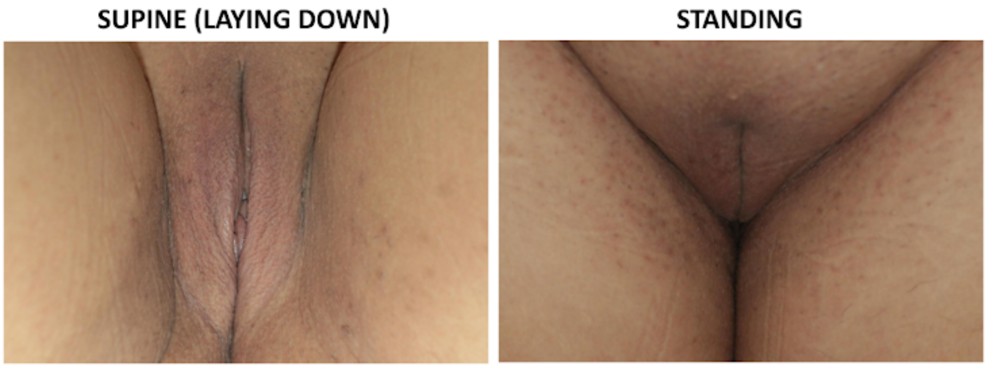

16. Has this person had vaginal cosmetic surgery? *

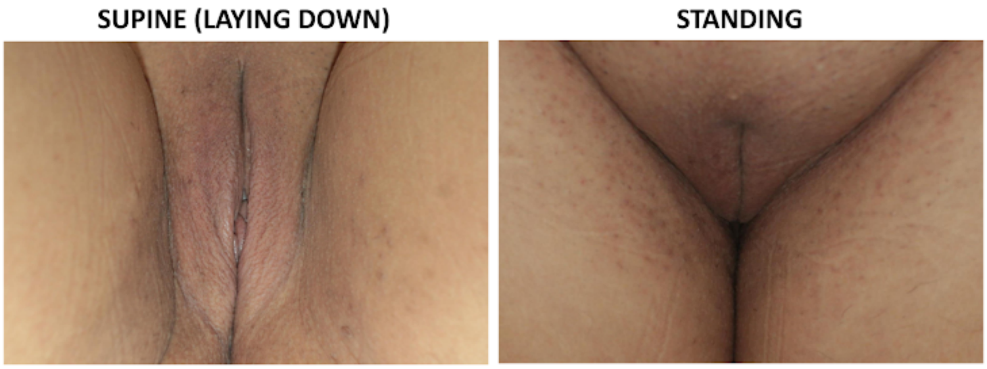

Mark only one oval.

- ( ) Yes
- ( ) No

17. Does this vagina look natural? *

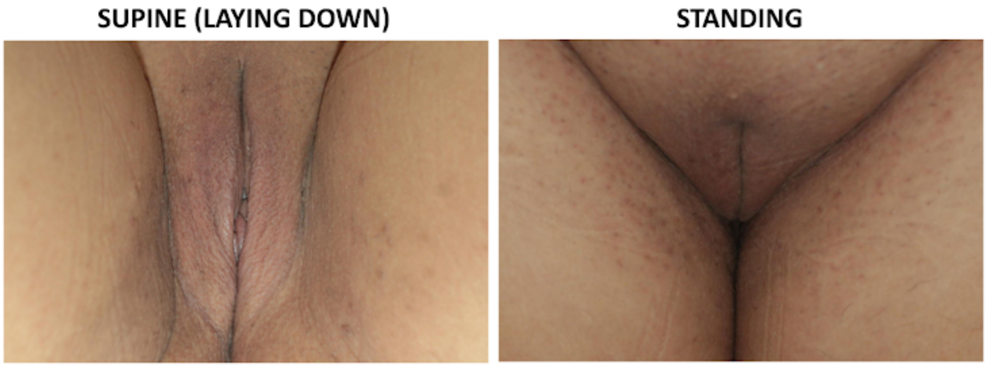

Mark only one oval.

	1	2	3	4	5	
Not	◯	◯	◯	◯	◯	Natural

18. Does this vagina look aesthetic (i.e. beautiful, cosmetic, attractive, or appealing)? *

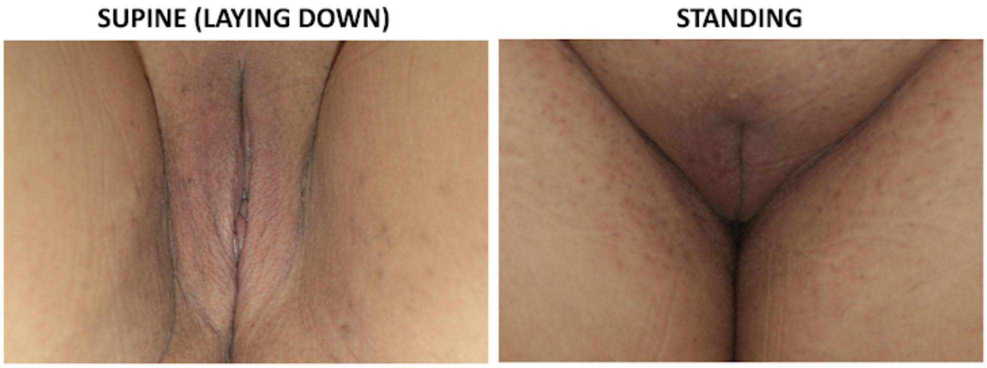

Mark only one oval.

	1	2	3	4	5	
Not	◯	◯	◯	◯	◯	Aesthetic

Picture 5.

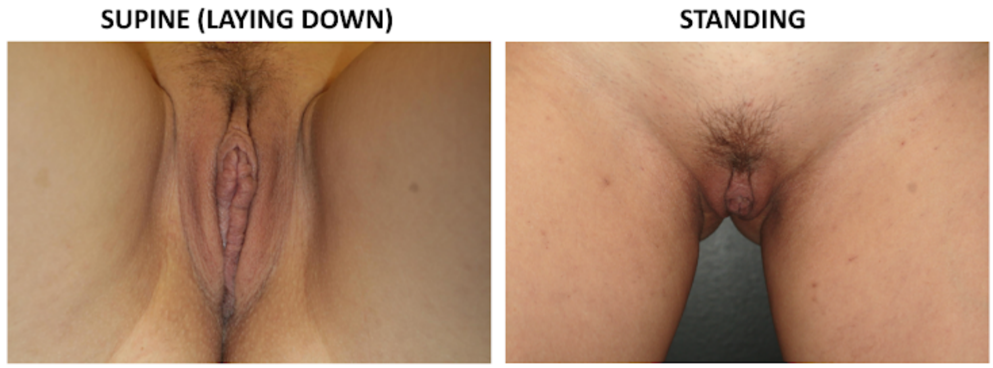

19. Has this person had vaginal cosmetic surgery? *

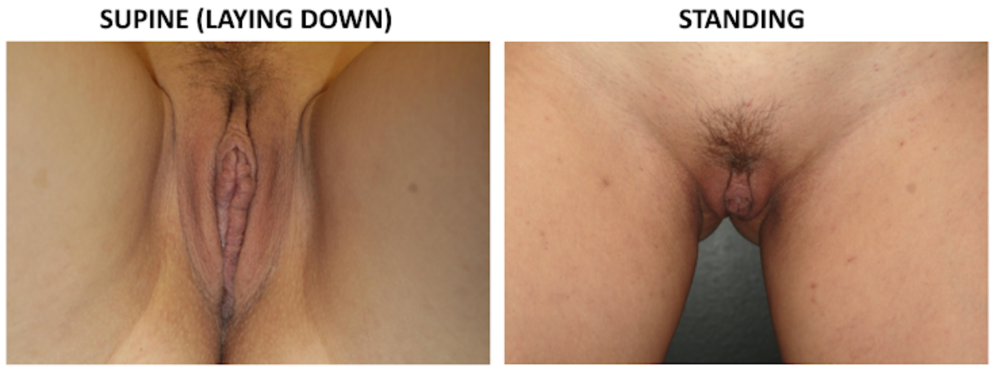

Mark only one oval.

- ( ) Yes
- ( ) No

20. Does this vagina look natural? *

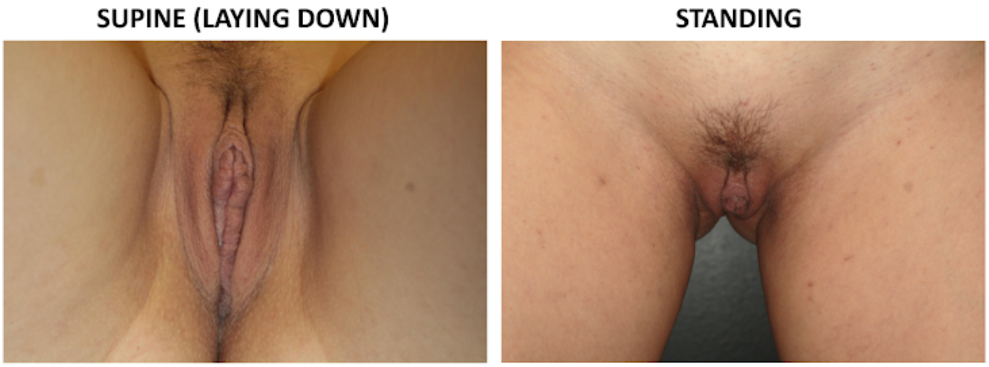

Mark only one oval.

	1	2	3	4	5	
Not	◯	◯	◯	◯	◯	Natural

21. Does this vagina look aesthetic (i.e. beautiful, cosmetic, attractive, or appealing)? *

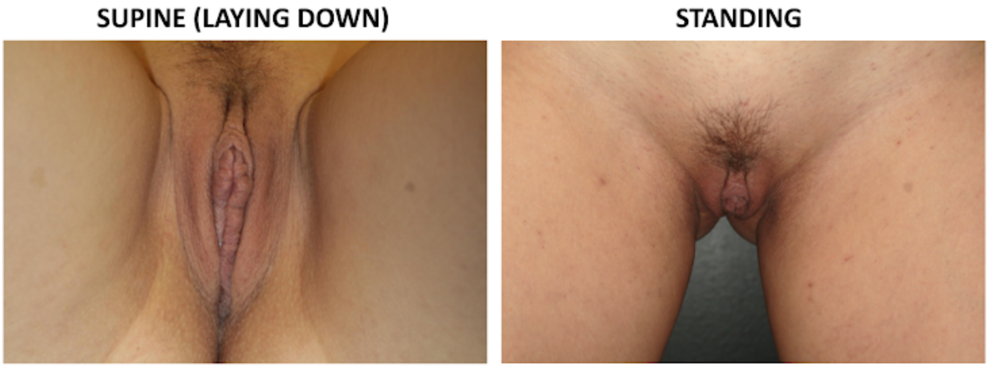

Mark only one oval.

	1	2	3	4	5	
Not	◯	◯	◯	◯	◯	Aesthetic

Picture 6.

SUPINE (LAYING DOWN)

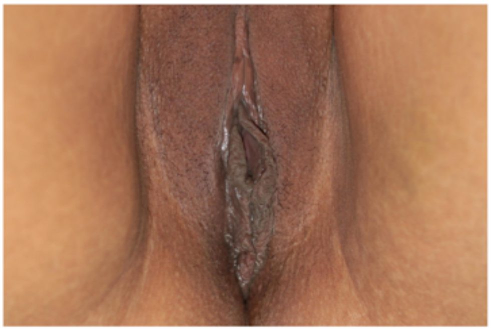

STANDING

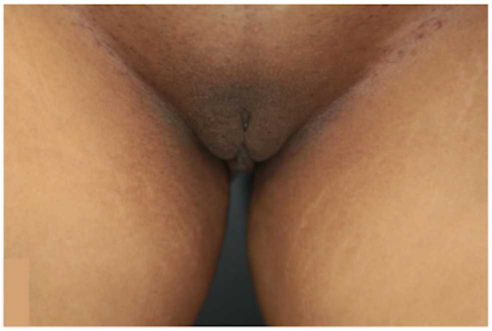

22. Has this person had vaginal cosmetic surgery? *

SUPINE (LAYING DOWN)

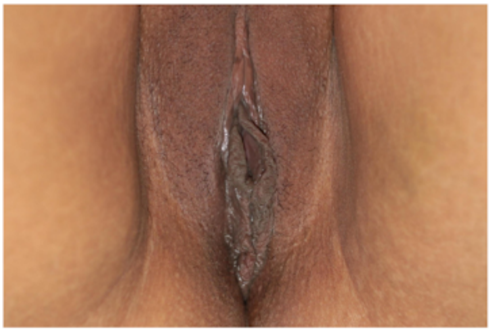

STANDING

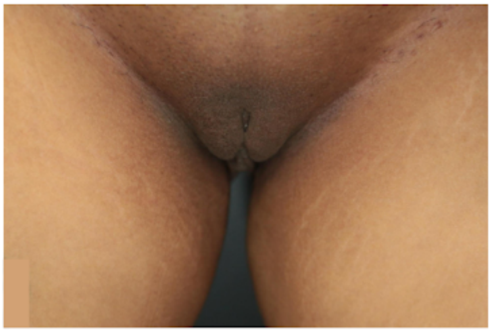

Mark only one oval.

- ( ) Yes
- ( ) No

23. Does this vagina look natural? *

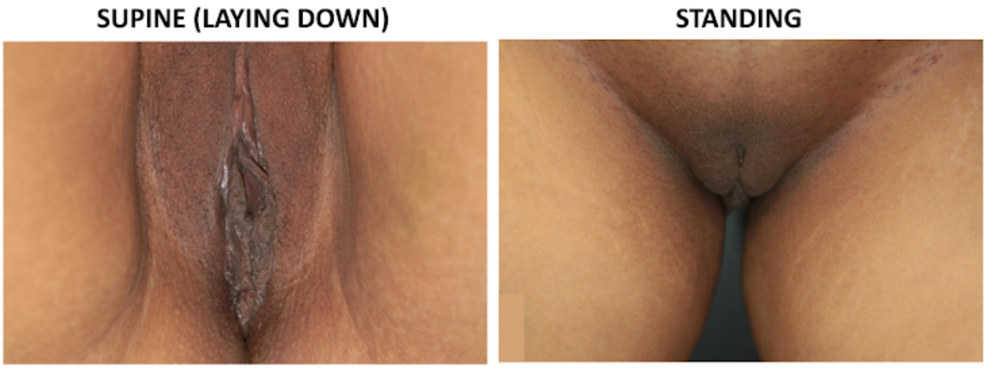

Mark only one oval.

	1	2	3	4	5	
Not	◯	◯	◯	◯	◯	Natural

24. Does this vagina look aesthetic (i.e. beautiful, cosmetic, attractive, or appealing)? *

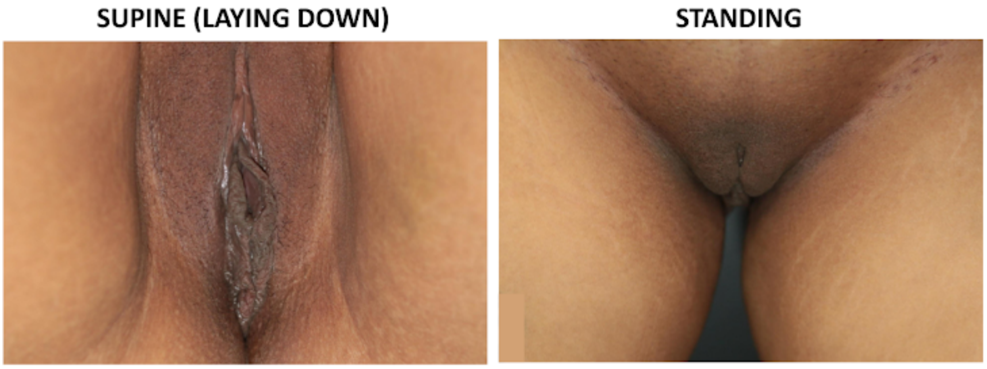

Mark only one oval.

	1	2	3	4	5	
Not	◯	◯	◯	◯	◯	Aesthetic

Picture 7.

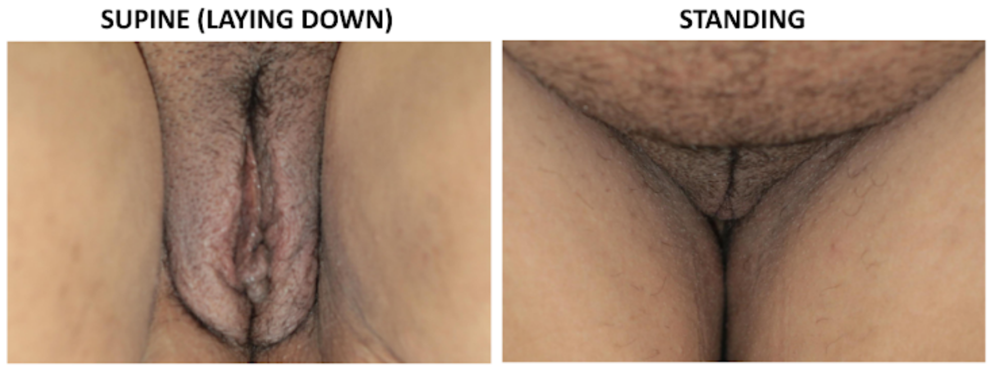

25. Has this person had vaginal cosmetic surgery? *

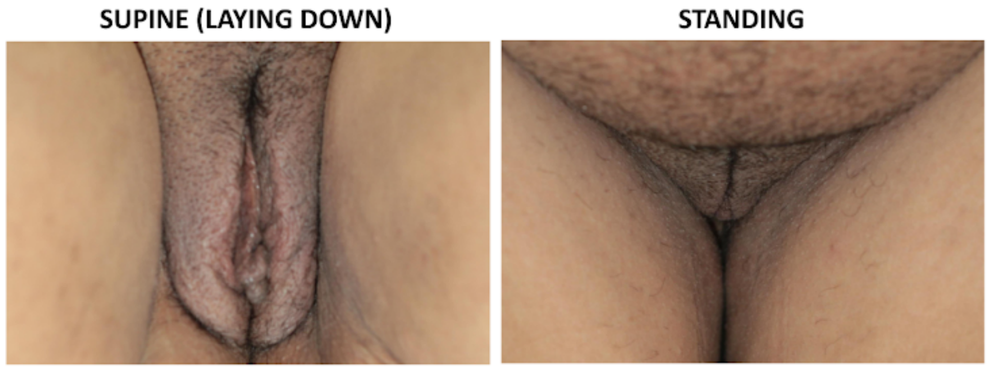

Mark only one oval.

- Yes
- No

26. Does this vagina look natural? *

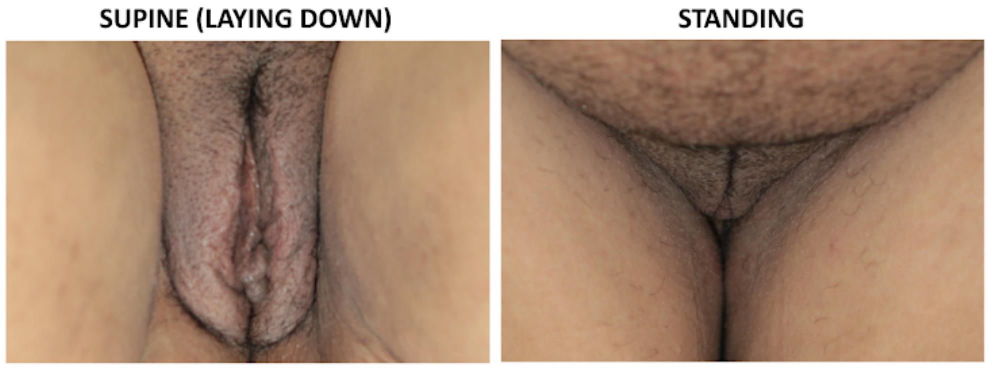

Mark only one oval.

	1	2	3	4	5	
Not	◯	◯	◯	◯	◯	Natural

27. Does this vagina look aesthetic (i.e. beautiful, cosmetic, attractive, or appealing)? *

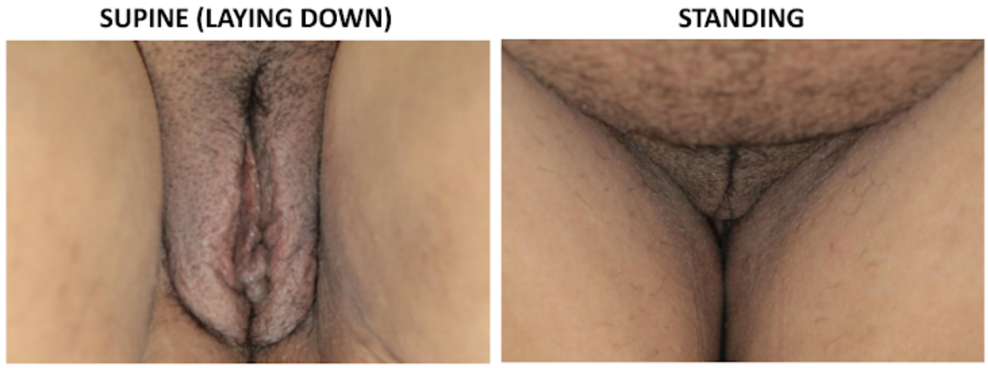

Mark only one oval.

	1	2	3	4	5	
Not	◯	◯	◯	◯	◯	Aesthetic

Picture 8.

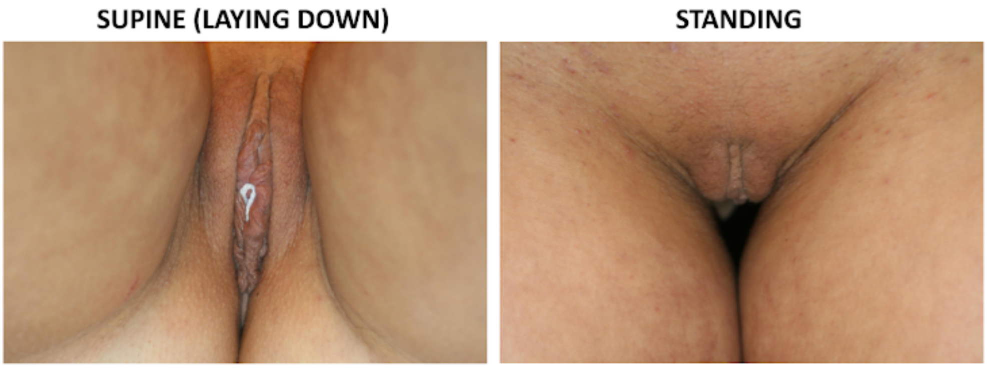

28. Has this person had vaginal cosmetic surgery? *

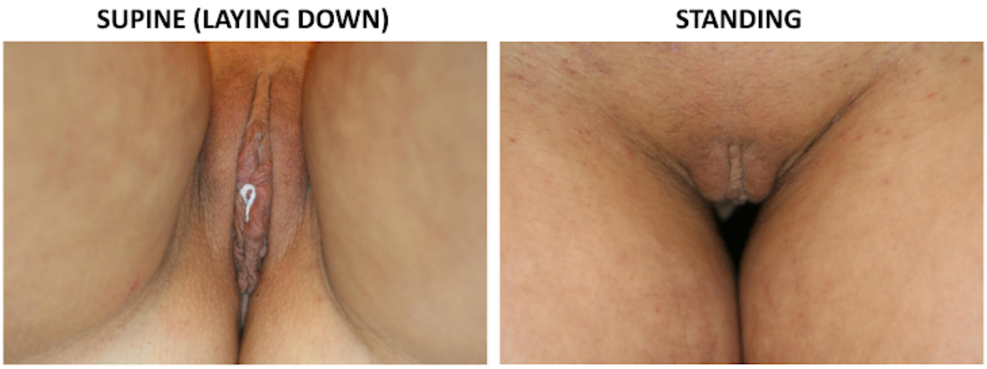

Mark only one oval.

- Yes
- No

29. Does this vagina look natural? *

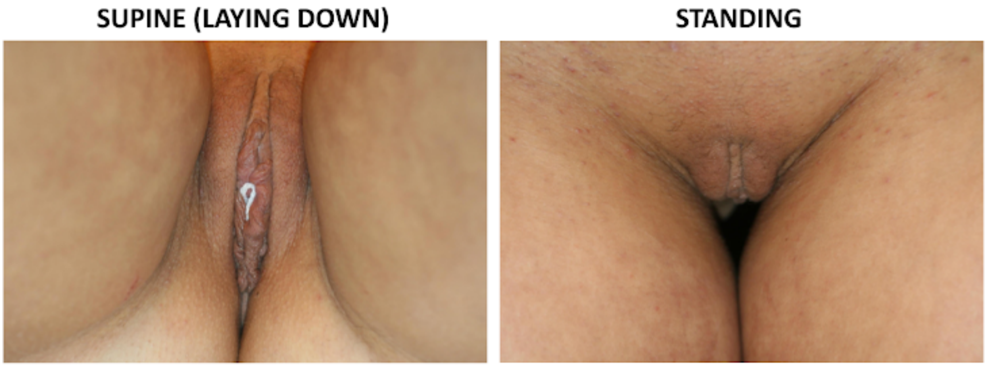

Mark only one oval.

	1	2	3	4	5	
Not	◯	◯	◯	◯	◯	Natural

30. Does this vagina look aesthetic (i.e. beautiful, cosmetic, attractive, or appealing)? *

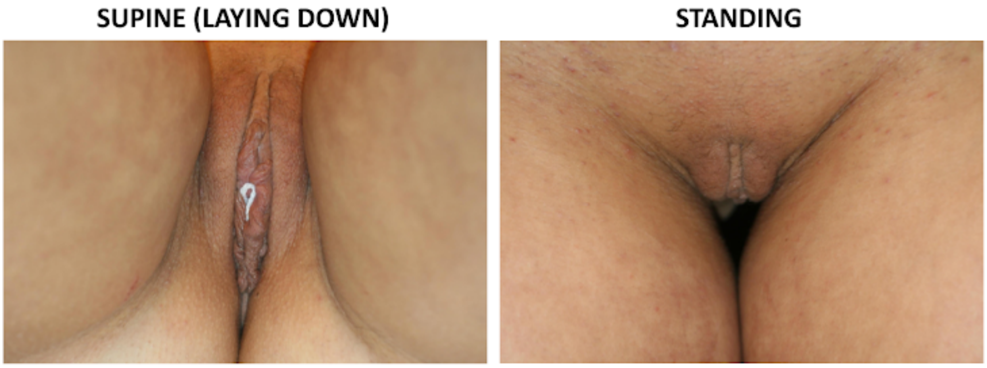

Mark only one oval.

	1	2	3	4	5	
Not	◯	◯	◯	◯	◯	Aesthetic

Picture 9.

SUPINE (LAYING DOWN) STANDING

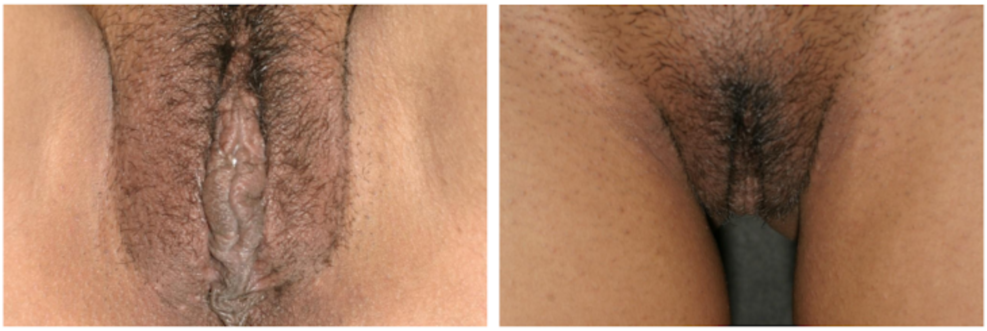

31. Has this person had vaginal cosmetic surgery? *

SUPINE (LAYING DOWN) STANDING

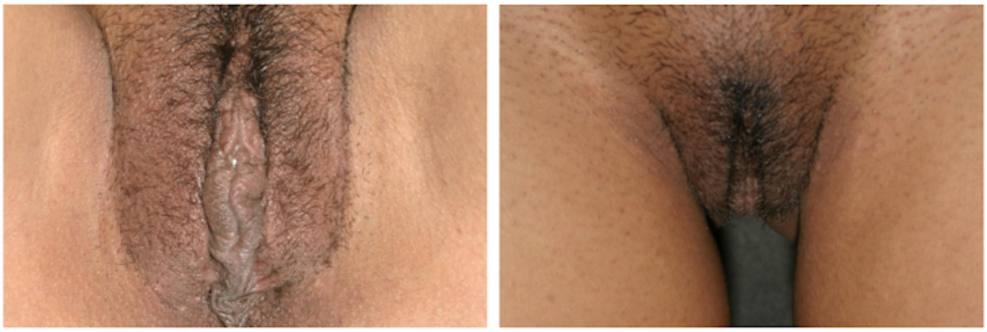

Mark only one oval.

- ( ) Yes
- ( ) No

32. Does this vagina look natural? *

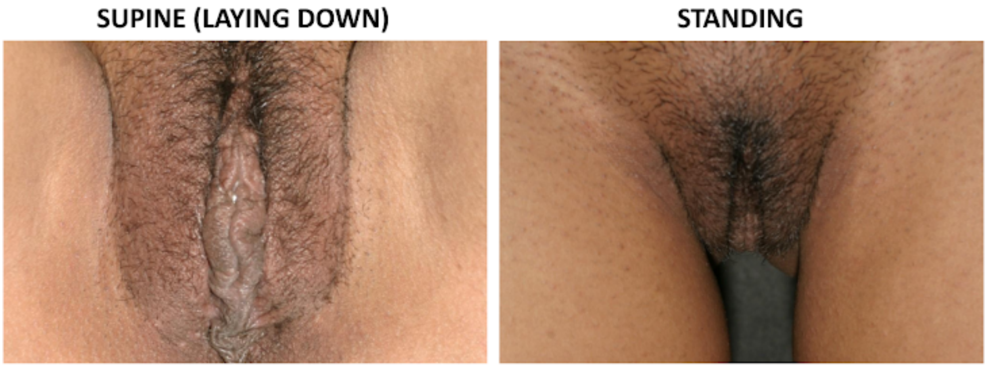

Mark only one oval.

	1	2	3	4	5	
Not	◯	◯	◯	◯	◯	Natural

33. Does this vagina look aesthetic (i.e. beautiful, cosmetic, attractive, or appealing)? *

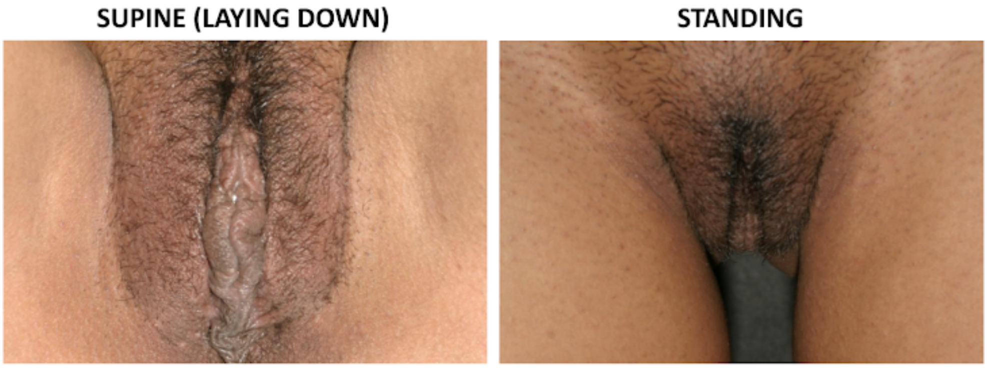

Mark only one oval.

	1	2	3	4	5	
Not	◯	◯	◯	◯	◯	Aesthetic

Picture 10.

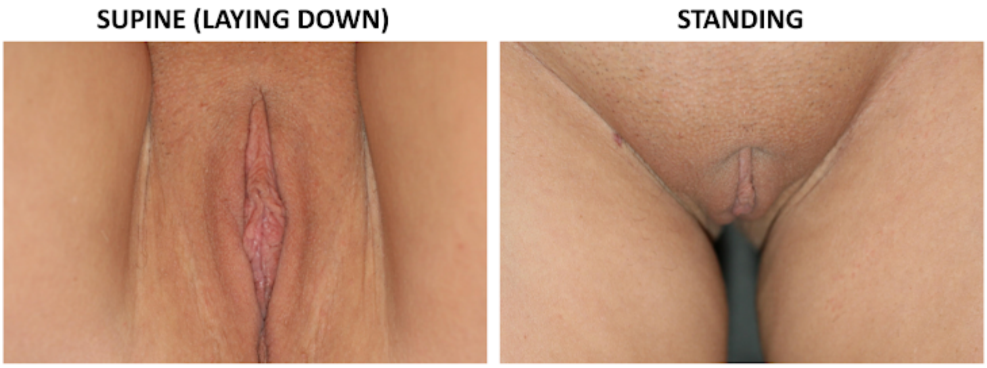

34. Has this person had vaginal cosmetic surgery? *

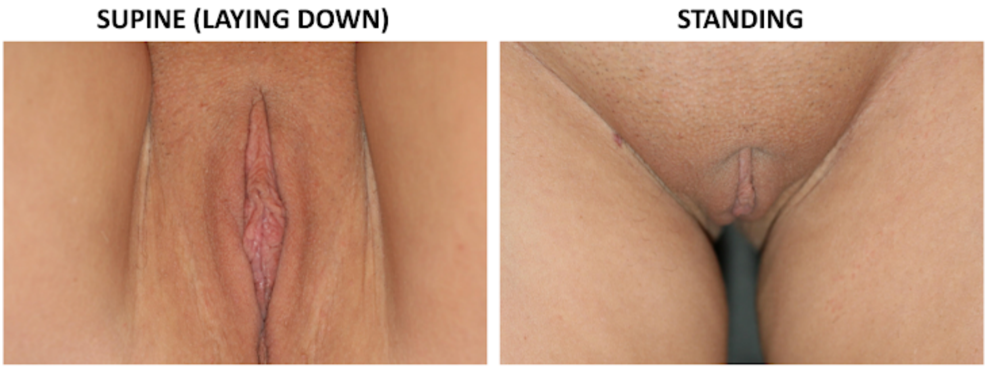

Mark only one oval.

- Yes
- No

35. Does this vagina look natural? *

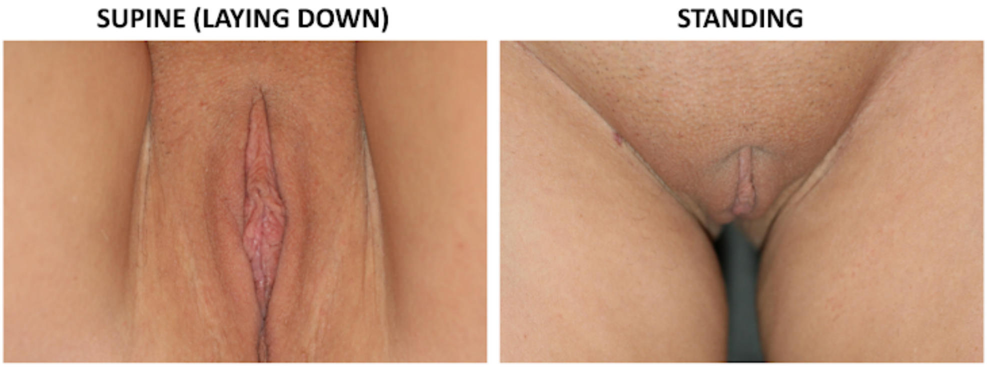

Mark only one oval.

	1	2	3	4	5	
Not	◯	◯	◯	◯	◯	Natural

36. Does this vagina look aesthetic (i.e. beautiful, cosmetic, attractive, or appealing)? *

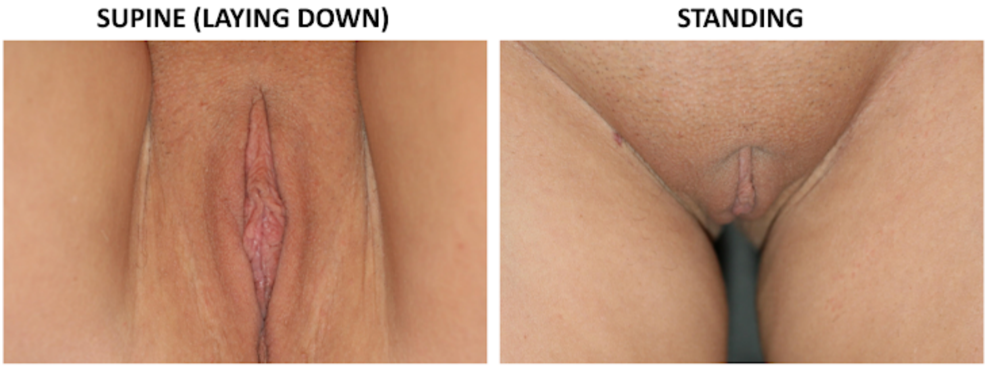

Mark only one oval.

	1	2	3	4	5	
Not	◯	◯	◯	◯	◯	Aesthetic

Final Questions

37. For the vaginas that you think had surgery, pick the reasons that helped you make this decision. *

Check all that apply.

- [ ] Too perfect
- [ ] Obvious scar
- [ ] Odd/Fake looking
- [ ] Looks transgender
- [ ] Looks like female genital mutilation (procedures that injure the vagina for non-medical or non-cosmetic purposes)
- [ ] Hair quality/type
- [ ] Aesthetic appearance
- [ ] Other: ______________________

38. For the vaginas that you think looked natural, pick the reasons that helped you make this decision. *

Check all that apply.

- [ ] Looks large
- [ ] Hair quality/type
- [ ] Aesthetic appearance
- [ ] Looks unoperated
- [ ] Other: ______________________

39. Please provide any feedback you may have regarding this study (optional).

This content is neither created nor endorsed by Google.

Google Forms
